# Adenosine A1 Receptors Participate in Excitability Changes after Cortical Epileptic Afterdischarges in Immature Rats

**DOI:** 10.3390/ph16121733

**Published:** 2023-12-15

**Authors:** Pavel Mareš, Libor Uttl, Martina Laczó, Zina BenSalem, Kateřina Vondráková, Petr Fábera, Grygoriy Tsenov, Hana Kubová

**Affiliations:** 1 Department of Developmental Epileptology, Institute of Physiology, Czech Academy of Sciences, 14200 Prague, Czech Republichana.kubova@fgu.cas.cz (H.K.); 2 National Institute of Mental Health, 25067 Klecany, Czech Republic; 3 Department of Neurology, Second Faculty of Medicine, Motol University Hospital, Charles University, 15006 Prague, Czech Republic

**Keywords:** adenosine receptors, cerebral cortex, electrical stimulation, epileptic afterdischarges, postictal period, rat, ontogeny

## Abstract

**Background**: Postictal refractoriness, i.e., the inability to elicit a new epileptic seizure immediately after the first one, is present in mature animals. Immature rats did not exhibit this refractoriness, and it is replaced by postictal potentiation. In addition to the immediate postictal potentiation, there is a delayed potentiation present at both ages. These phenomena were studied using cortical epileptic afterdischarges as a model. **Objective**: We aimed to analyze participation of adenosine A1 receptors in postictal potentiation and depression. **Methods**: Adenosine A1 receptors were studied by means of Western blotting in the cerebral cortex with a focus on the age groups studied electrophysiologically. Stimulation and recording electrodes were implanted epidurally in 12- and 25-day-old rats. The first stimulation always induced conditioning epileptic afterdischarge (AD), and 1 min after its end, the stimulation was repeated to elicit the second, testing AD. Then, the drugs were administered and paired stimulations were repeated 10 min later. A selective agonist CCPA (0.5 and 1 mg/kg i.p.) and a selective antagonist DPCPX (0.1, 0.5 and 1 mg/kg i.p.) were used to examine the possible participation of adenosine A1 receptors. **Results**: Control younger animals exhibited potentiation of the testing AD and a moderate increase in both conditioning and testing ADs after an injection of saline. The A1 receptor agonist CCPA shortened both post-drug ADs, and neither potentiation was present. The administration of an antagonist DPCPX resulted in marked prolongation of the conditioning AD (delayed potentiation), and the second testing AD was shorter than the post-drug conditioning AD, i.e., there was no longer immediate potentiation of ADs. To eliminate effects of the solvent dimethylsulfoxide, we added experiments with DPCPX suspended with the help of Tween 80. The results were similar, only the prolongation of ADs was not as large, and the testing ADs were significantly depressed. The older control group exhibited a nearly complete suppression of the first testing AD. There was no significant change in the conditioning and testing ADs after CCPA (delayed potentiation was blocked). Both groups of DPCPX-treated rats (with DMSO or Tween) exhibited significant augmentation of delayed potentiation but no significant difference in the immediate depression. Adenosine A1 receptors were present in the cerebral cortex of both age groups, and their quantity was higher in 12- than in 25-day-old animals. **Conclusions**: An agonist of the A1 receptor CCPA suppressed both types of postictal potentiation in 12-day-old rats, whereas the A1 antagonist DPCPX suppressed immediate potentiation but markedly augmented the delayed one. Immediate postictal refractoriness in 25-day-old rats was only moderately (non-significantly) affected; meanwhile, the delayed potentiation was strongly augmented.

## 1. Introduction

Adenosine plays a role in many physiological processes as a powerful inhibitory modulator [1]. It is now taken as a potential drug for the treatment of some pathologies [2]. It acts on four types of metabotropic receptors (A1, A2A, A2B, A3) coupled with G proteins. Two types of these receptors are predominant in the brain [3,4]—the nearly ubiquitous inhibitory A1 (with the highest concentration in the cortex and hippocampus) and the second type, excitatory A2A, present mainly but not exclusively in the basal ganglia [2,5]. Among other roles [6], the adenosinergic system plays an important role in epilepsy [7] and especially in the arrest of seizures. Adenosine may be taken as an endogenous anticonvulsant [8]. This role was demonstrated not only in animal models of seizures—e.g., [9,10]—but also in human tissue where inhibitory A1 receptors participate in the arrest of seizures and postictal depression [11]. In animal models, potentiation of the A1 receptor action has a marked anticonvulsant effect, and a selective antagonist DPCPX is strongly proconvulsant [10,12].

Many types of epileptic seizures are followed by a period of postictal depression. Among other changes, postictal refractoriness, i.e., a failure to elicit a new seizure immediately after the end of the first epileptic seizure, participates in a pattern of postictal depression. This refractoriness might be taken as an expression of the overlasting activation of mechanisms arresting seizures. Inhibitory systems are probably responsible for both the arrest of seizures and postictal refractoriness. The first proof was presented with an antagonist of opioid mu receptors naloxone—it was found to suppress postictal refractoriness in the amygdala [13] and in the hippocampus [14] of adult rodents. Our older data demonstrated that these mechanisms may not be the same in different types of seizures generated in various brain structures. The action of naloxone on hippocampal epileptic afterdischarges was confirmed, but the refractoriness after maximal electroshock seizures was not affected [15]. Therefore, we started to study another model where both postictal potentiation and depression according to age are present—cortical epileptic afterdischarges elicited by low-frequency rhythmic stimulation of the sensorimotor region of the cerebral cortex. These afterdischarges are characterized by a spike-and-wave rhythm in the EEG and clonic seizures of forelimb and head muscles. Spike-and-wave rhythmic activity is generated by cortico-thalamo-cortical mechanisms [16]; in the case of cortical afterdischarges, this activity must spread into the motor system to generate clonic seizures. Cortical inhibitory systems are complex [17], and therefore, we started to study other neurotransmitter systems possibly involved in the postictal refractoriness in the cerebral cortex. 

Both competitive and noncompetitive antagonists of the GABA_A_ supramolecular complex failed to affect cortical postictal refractoriness, whereas antagonists of GABA_B_ receptors were found to partially suppress this phenomenon [18,19]. Therefore, we started to study the possible role of other inhibitory systems. The adenosinergic modulatory inhibitory system was chosen as the first because of its role in the arrest of seizures [9,10,11]. The role of the A1 type of adenosine receptors was analyzed using a selective agonist and antagonist. Rats aged 25 days old were used because they exhibit the same postictal refractoriness as adult animals [20].

Postictal refractoriness is not present during early developmental stages. It appears and matures during the postnatal development of rats [20]. Therefore, we extended the study to a developmental stage where there is immediate postictal potentiation instead of postictal refractoriness—rats aged 12 days old—to find if there is a possibility to establish refractoriness at this stage when it is not yet present and if the A1 receptor agonist is able to suppress the potentiation. In addition to the immediate postictal potentiation during early postnatal brain development, there is a delayed potentiation (around 10 min after the seizure) in both age groups studied, which is better expressed in the younger group. Postictal potentiation might have an important role in the high excitability of the immature brain and the failure of seizure arrest and thus an easy generation of status epilepticus in the pediatric population. 

In addition to electrophysiological experiments, we studied the presence of A1 receptors in the cerebral cortex of developing rats. 

We hypothesized that the activation of adenosine A1 receptors might suppress both immediate and delayed postictal potentiation. An antagonist of A1 receptors might increase both types of postictal potentiation in 12-day-old rat pups and suppress postictal depression in 25-day-old rats.

## 2. Results

### 2.1. Development of Adenosine A1 Receptors in the Cerebral Cortex

**Figure 1:**
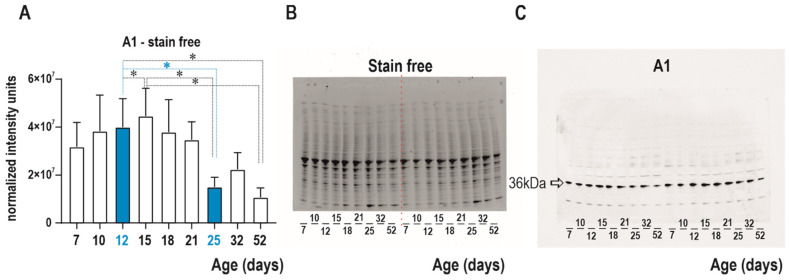
Western blot analysis of A1 receptor density in cortical homogenate in nine age groups. (**A**) shows a bar graph of A1 receptor density in the cortex (mean normalized intensity units + SEM). Age groups PD 12 and PD 25 are marked in blue for better clarity. Abscissae from left to right: 7-, 10-, 12-, 15-, 18-, 21-, 25-, 32-, and 52-day-old rats. Asterisks denote significant differences versus the 12-day-old group of animals with *p*-values of <0.05. (**B**) Stain-free blot visualization of total protein concentration on membrane was performed using stain-free technology from Bio-Rad and used for normalization. (**C**) shows the density of the target A1 receptor (36 kDa) visualized using a chemiluminescent substrate during brain development in control animals. Abscissae from left to right: 7-, 10-, 12-, 15-, 18-, 21-, 25-, 32-, and 52-day-old rats in identical replicates.

A1 receptors were demonstrated in all studied age groups (7 to 52 days). The maximum intensity was found in the second and third weeks, then a steep decrease was found in both stain-free and normalized data. The density of these receptors was significantly lower (50%) at postnatal day (PD) 25 compared to 12-day-old rats (PD 25 rats 49.56%± 8.917%, and 100.0% ± 10.28% in PD12 animals, *p* = 0.0008) if the stain-free technique was used (Figure 1).

### 2.2. Electrophysiological Experiments

#### 2.2.1. Effects of Solvents

**Figure 2:**
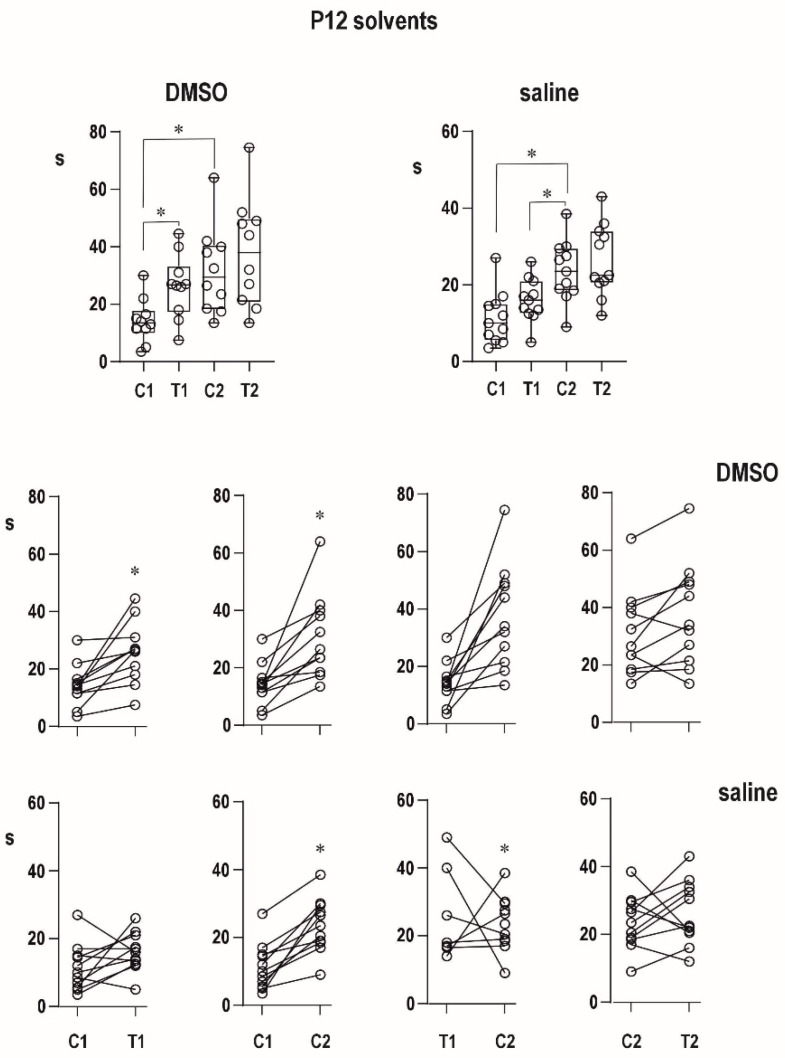
Effect of solvents on ADs duration in PD12 rats. Upper row—effect of dimethylsulfoxide (DMSO, left) and effect of saline (right). In addition to boxes demonstrating median with 95% CI, circles demonstrate individual values. Middle row-comparison of duration between the two stimulations. From left to right: C1 to T1; C1 to C2; T1 to C2; C2 to T2. Lower row—the same for saline controls. Asterisks denote significant difference between individual afterdischarges.

**Figure 3:**
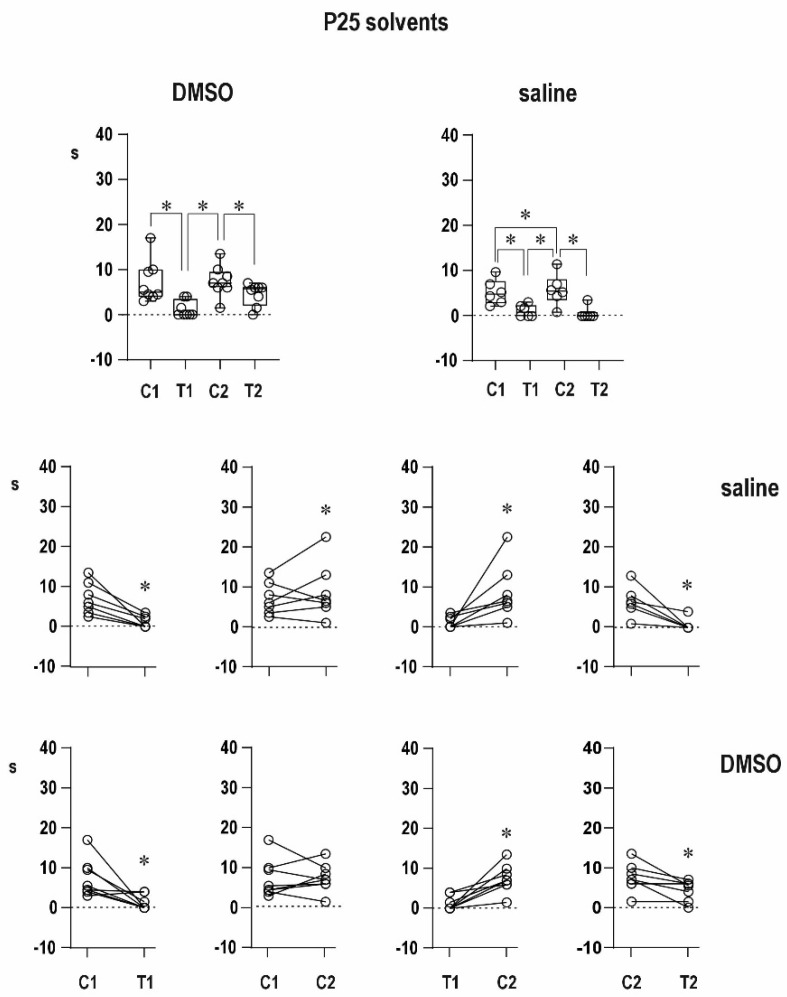
Effect of solvents on ADs duration in P25 rats. All details as in Figure 2.

Neither saline nor 50% dimethylsulfoxide significantly changed the immediate postictal potentiation (prolongation of ADs) in 12-day-old rat pups as well as delayed potentiation in both age groups. Postictal depression in 25-day-old rats also remained unaffected (Figure 2 and Figure 3).

#### 2.2.2. Agonist of A1 Adenosine Receptors CCPA 

##### Twelve-Day-Old Rats (Figure 4)

Pre-drug conditioning ADs lasted on the average 10.8 ± 0.7 s in the control group and 9.4 ± 1.7 and 8.3 ± 1.9 s, respectively, in the two drug groups. A significantly longer testing AD was present in all three groups, and its duration varied from 23.6 ± 1.3 s in control rats to 20.1 ± 2.4 and 19.1 ± 3.1 s, respectively, in the two other groups.

The post-drug conditioning AD was significantly longer than the pre-drug one in controls (19.7 ± 4.4 s) but not in the experimental animals—12.1 ± 2.5 and 6.6 ± 1.1 s, respectively, in the 0.5- and 1-mg/kg groups—and a shortening of the duration of this AD reached the level of statistical significance only after the 1-mg/kg dose. Testing ADs were again present in all animals and lasted 24.4 ± 4.6 s in the control group and 13.5 ± 3.6 and 5.7 ± 1.2 s, respectively, in the two CCPA groups. The potentiation of the testing ADs was abolished by either dose of CCPA, but the shortening of ADs did not reach the level of statistical significance. Similarly to conditioning afterdischarges, either dose of CCPA resulted in a significantly shorter testing AD in comparison with the corresponding pre-drug ones.

The higher dose (1 mg/kg) was able to completely suppress the immediate postictal potentiation. Only a faint tendency to suppress the delayed potentiation was seen.

**Figure 4:**
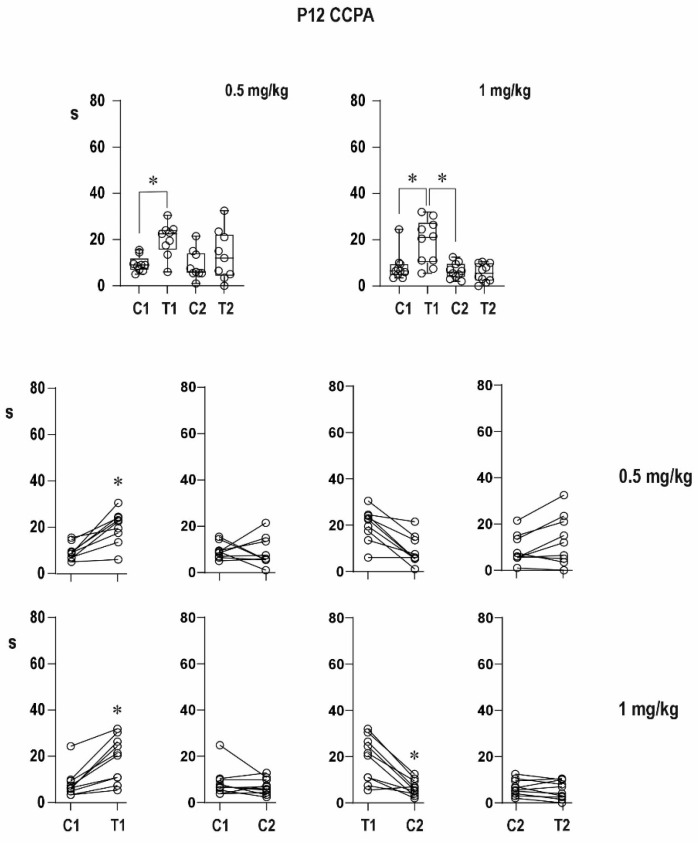
Effect of two doses of CCPA on AD duration in 12-day-old rats. Details as in Figure 2; only doses of CCPA are presented with individual graphs or rows.

#### 2.2.3. Agonist of A1 Adenosine Receptors CCPA 

##### Twenty Five-Day-Old Rats (Figure 5)

Pre-drug conditioning ADs did not differ among the three groups—7.1 ± 1.5 s in controls and 7.8 ± 1.3 and 8.5 ± 1.0 s in the two drug groups, respectively. Pre-drug testing ADs were recorded in some of the animals (in 3 out of 7 controls and in 7 out of 11 and 5 out of 10 in the two experimental groups). The duration of ADs in the control group (1.1 ± 0.6 s), did not reach the level of significance (*p* = 0.0576), but ADs in the two CCPA groups were significantly shorter than those of the corresponding conditioning pre-drug ADs (1.0 ± 0.3 and 1.5 ± 0.5 s, respectively).

Post-drug groups exhibited conditioning ADs lasting 8.9 ± 2.6 s in the control group (*p* = 0.576) and significantly shorter (7.5 ± 1.4 and 6.3 ± 1.6 s, respectively) in the two CCPA groups. The incidence of the testing ADs did not significantly differ from pre-drug data (in 2 out of 7 controls and in 6 out of 11 and 5 out of 10 in the two experimental groups). Again, as with conditioning ADs, the duration of testing ADs was shorter than that of the conditioning ones in CCPA groups (1.5 ± 0.6 and 1.1 ± 0.4 s) but not in controls (0.9 ± 0.6 s, *p* = 0.0637) and comparable with the pre-drug testing ADs. 

The transition to mixed AD was never seen after CCPA administration in any age and dose group.

The effect of CCPA was only marginal in this age group. 

**Figure 5:**
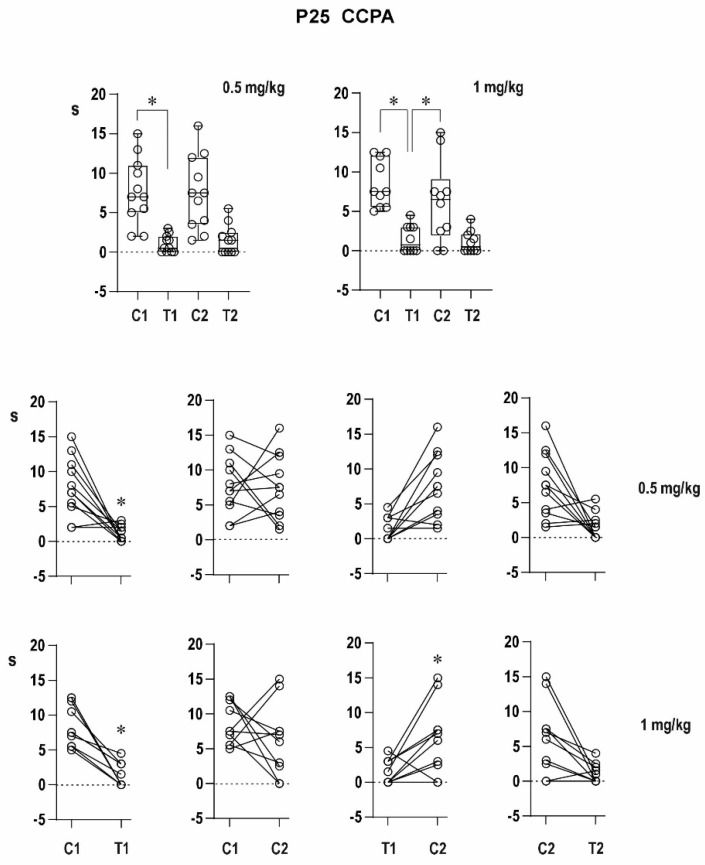
Effect of two doses of CCPA on AD duration in 25-day-old rats. Details as in Figure 2; doses of CCPA are again presented with individual graphs or rows.

#### 2.2.4. Antagonist of Adenosine A1 Receptors DPCPX 

##### Twelve-Day-Old Rats (Figure 6)

Outlined differences among pre-drug conditioning ADs did not reach the level of statistical significance (10.4 ± 2.1, 8.3 ± 0.8, 8.6 ± 1.4, and 11.1 ± 2.1 s, respectively). All four 12-day-old groups exhibited testing ADs significantly longer than the conditioning one (24.9 ± 2.1, 23.9 ± 2.3, 25.6 ± 3.6, and 26.3 ± 3.3 s, respectively). Mixed ADs, i.e., a transition to the limbic type of AD characterized by the same EEG pattern and behavior (automatisms instead of clonic seizures) as ADs elicited by hippocampal stimulation, were never seen in control DMSO rats and animals before the 0.1-mg/kg dose administration but in three out of nine and one out of nine rats in the groups administered with two higher doses of DPCPX.

The post-drug DMSO control and the three drug groups exhibited significantly longer conditioning ADs than before injection (56.9 ± 5.8, 107.8 ± 21.5, 177.8 ± 24.9, and 129.2 ± 65.2 s), i.e., delayed potentiation was augmented. Testing ADs tended to be shorter than post-drug conditioning ones (44.4 ± 6.4, 97.5 ± 16.9, 120.0 ± 22.9, and 71.2 ± 17.3 s), i.e., immediate potentiation was abolished, but postictal depression was not established. The testing ADs were significantly longer than pre-drug testing ADs. A transition to the limbic type of ADs was exhibited in 3 out of 10 animals in the DMSO-treated group, 5 out of 13, all 9, and 3 out of 9 in the three DPCPX groups of rats, respectively.

There was a marked augmentation of the delayed potentiation, and the immediate one was blocked but not reversed to depression.

**Figure 6:**
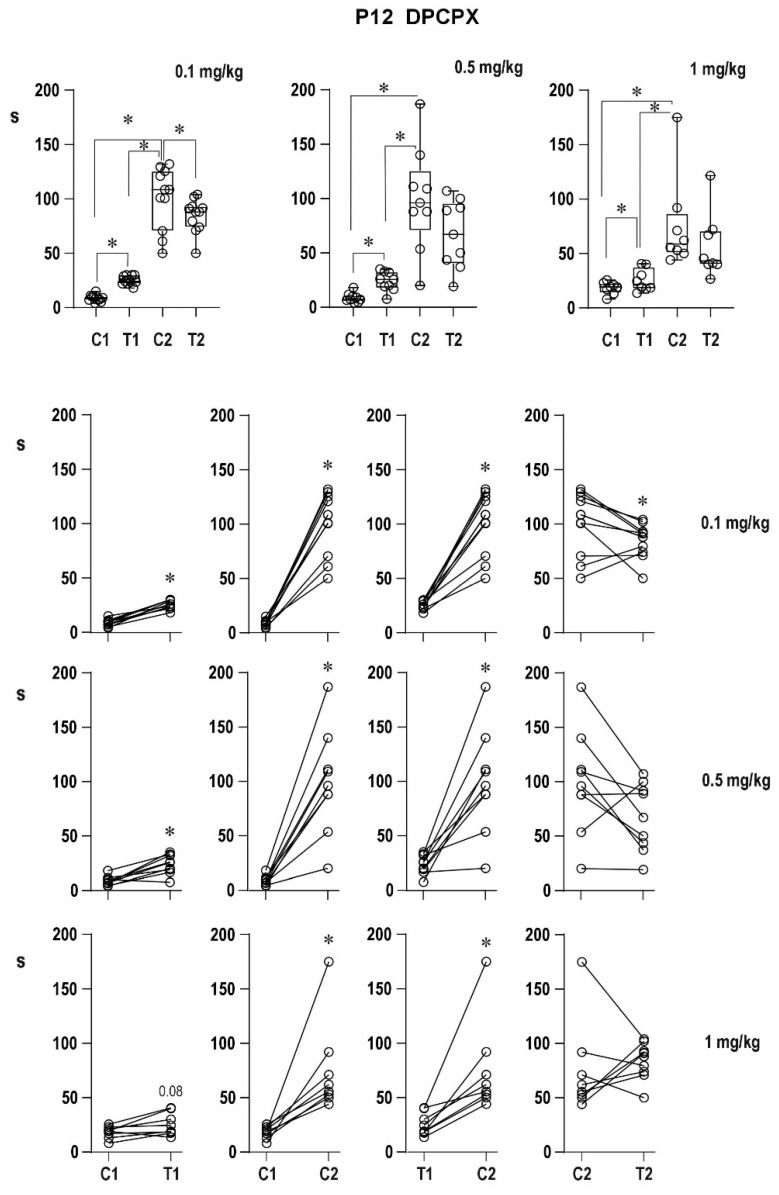
Effect of three doses of DPCPX dissolved in DMSO on AD duration in 12-day-old rats. Details as in Figure 2; doses of DPCPX are again presented with individual graphs or rows.

##### Twenty-Five-Day-Old Rats (Figure 7)

Pre-drug conditioning ADs did not significantly differ among the four groups (7.3 ± 1.7 s in the DMSO group and 8.0 ± 1.0, 11.1 ± 1.5, and 7.6 ± 1.8 s, respectively, in the three experimental groups). The same (i.e., no significant differences among groups) was true for pre-drug testing ADs. Immediate postictal depression was not significant in controls in contrast to the three drug groups, where *p* was smaller than 0.05.

Post-drug conditioning control ADs were not prolonged after DMSO administration (7.4 ± 1.2 s), and the duration of testing ADs in the control group did not significantly differ from pre-DMSO testing ADs. DPCPX resulted in significantly prolonged conditioning ADs (35.6 ± 6.5, 77.1 ± 9.8 and 13.2 ± 1.5 s), and testing ADs (which appeared in 2 out of 7, 9 out of 10, and 11 out of 13 rats) lasted 2.8 ± 1.2, 3.0 ± 1.0, and 5.3 ± 1.9 s in the three DPCPX groups, respectively, i.e., a significant shortening was present. The transition to mixed AD was recorded in this age group—in 2 out of 7 animals after the 0.1-mg/kg dose, 9 out of 10 after the 0.5-mg/kg dose, and in 2 out of 11 after the highest dose. All changes were more marked after the 0.1- and 0.5-mg/kg doses than after the highest dose (1 mg/kg).

Delayed postictal potentiation was strongly augmented in both age groups, and immediate potentiation in 12-day-old rats was suppressed.

**Figure 7:**
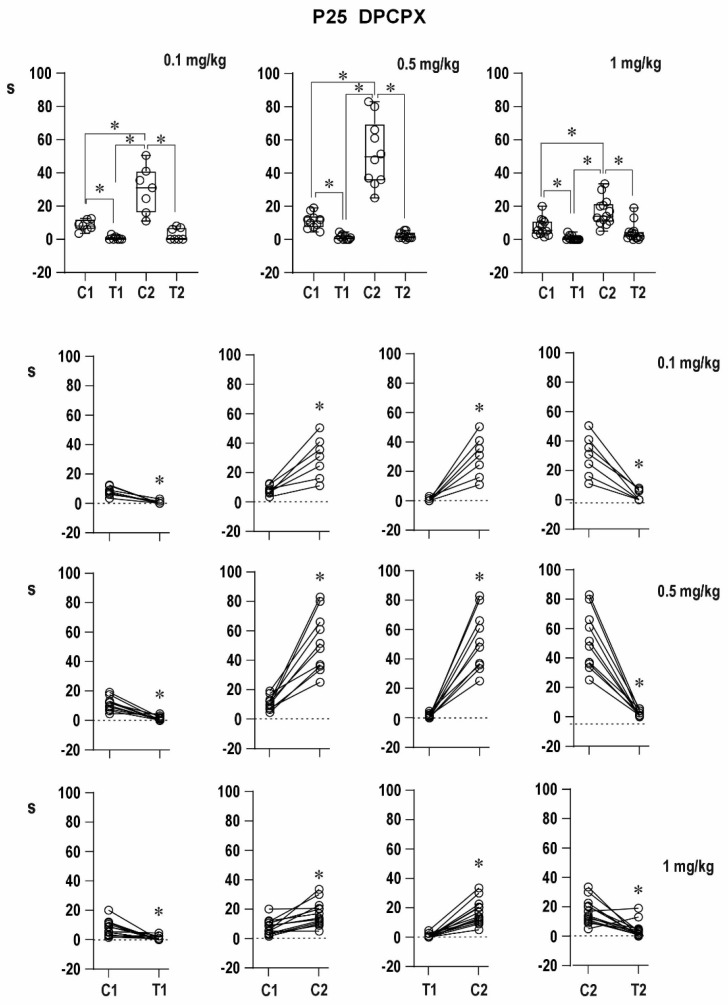
Effect of three doses of DPCPX dissolved in DMSO on AD duration in 25-day-old rats. Details as in Figure 2; doses of DPCPX are again presented with individual graphs or rows.

##### Twelve-Day-Old Rats—DPCPX in Suspension (Figure 8)

The pre-drug conditioning AD in control rats was the same as in the pre-DMSO group, and its duration was 10.7 ± 0.8 s. The two dose groups (0.5 and 1 mg/kg) exhibited these ADs as 15.1 ± 2.6 and 16.6 ± 2.8 s long, respectively. Testing pre-drug ADs lasted significantly longer than conditioning ADs—23.6 ± 1.3, 28.9 ± 8.4, and 28.2 ± 5.0 s, respectively, in the control and two experimental groups.

The duration of the two post-DPCPX conditioning ADs was markedly prolonged to 146.7 ± 48.2 and 175.9 ± 78.2 s, respectively, compared to 19.7 ± 4.4 s in controls, i.e., delayed potentiation was increased. On the other hand, testing ADs lasted 24.4 ± 4.6 s in the control group and 70.4 ± 9.2 and 78.5 ± 15.7 s in the two DPCPX groups, i.e., either dose of DPCPX led to a significant prolongation of both conditioning and testing ADs, but in spite of this, the prolongation of immediate postictal potentiation was lost. On the contrary, the 1-mg/kg dose led to a depression of the testing AD. The presence of the mixed type of ADs was observed only after the two doses of DPCPX in one and three animals out of eight, respectively.

**Figure 8:**
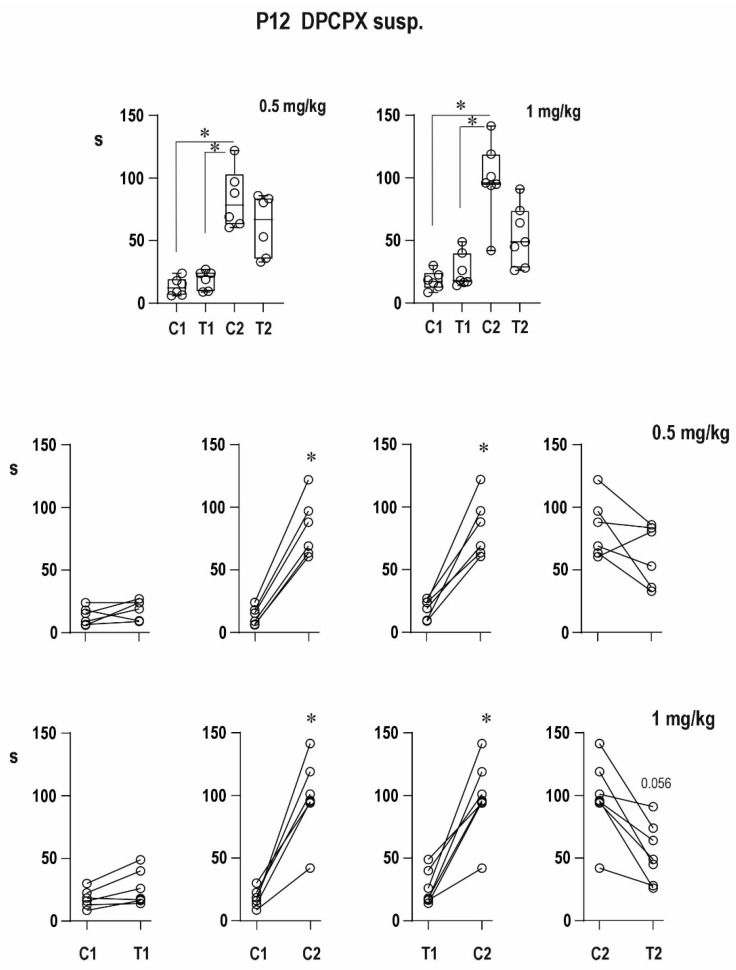
Effect of two doses of DPCPX in suspension on AD duration in 12-day-old rats. All details as in Figure 2 and Figure 6.

##### Twenty Five-Day-Old Rats—DPCPX in Suspension (Figure 9)

Pre-drug conditioning ADs lasted 7.1 ± 1.5, 12.4 ± 2.4, and 18.6 ± 5.5 s, respectively, in the three groups. Due to the high variability of data in the DMSO groups, the difference among groups did not reach the level of statistical significance. Testing ADs were present in three out of seven control animals, and their average duration was 1.1 ± 0.6 s. The two drug groups exhibited testing ADs in two out of eight and three out of eight cases with a mean duration of 0.6 ± 0.4 and 0.6 ± 0.3 s, respectively. The difference between conditioning and testing ADs was significant in the 0.5-mg/kg group.

Post-DPCPX conditioning ADs lasted longer in the two experimental groups (31.4 ± 6.4 and 33.5 ± 7.5 s, respectively) than in the control animals with an average duration of 8.9 ± 2.6 s. Delayed potentiation tended to be augmented even by this formula of DPCPX. Testing ADs were present in two out of seven controls and in five out of eight rats in both DPCPX groups. Their average duration was 0.9 ± 0.6 s in controls and 1.9 ± 0.8 and 5.6 ± 2.9 s in the 0.5- and 1-mg/kg groups, respectively. The differences among the three groups in the duration of testing ADs did not reach the level of statistical significance, but the testing AD after the 1-mg/kg dose was significantly longer than the corresponding pre-drug one. The transition of testing ADs from spike-and-wave to the mixed type was present in two out of seven controls and three out of eight rats after the 0.5-mg/kg dose but in none of the animals with the higher dose. 

The results were analogous to DPCPX dissolved in DMSO, i.e., it markedly increased the delayed potentiation in both age groups, the immediate potentiation in 12-day-old rats was suppressed with a tendency to depression after the 1-mg/kg dose, and unchanged postictal depression in 25-day-old rats. 

**Figure 9:**
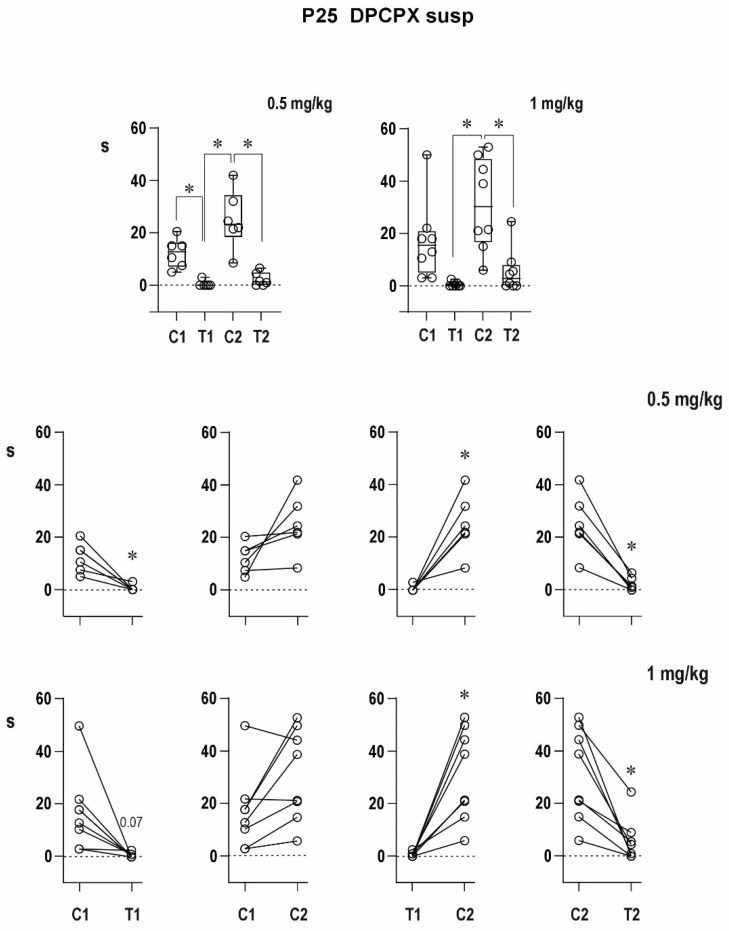
Effect of two doses of DPCPX in suspension on AD duration in 25-day-old rats. All details as in Figure 2 and Figure 7.

## 3. Discussion

Immediately after seizures, postictal depression appears in the mature brain. A part of this depression is refractoriness, i.e., the inability to elicit a new seizure. We demonstrated that at early stages of maturation, seizures are followed by postictal potentiation, i.e., the same epileptogenic insult induces seizures longer than the first one [20]. Control animals in the present experiment confirmed our previously published data—a presence of immediate potentiation instead of refractoriness in 12-day-old rats and immediate postictal refractoriness in 25-day-old animals [20]. The potentiation in the younger age group might be due to the overexpression of NMDA and AMPA receptors at the end of the second postnatal week combined with the immaturity of inhibitory systems—see schematic drawing in the paper of Jensen [21]. In addition to the immediate changes in excitability, there are also delayed changes in excitability—again, potentiation which is marked in 12-day-old rats but it is present also in 25-day-old animals.

An organic solvent dimethylsulfoxide diluted to 50% diminished progressive prolongation of ADs in the second pair in 12-day-old animals, and it tended to attenuate postictal refractoriness in the 25-day-old group. It might indicate a possible proconvulsant action of this solvent in the model of paired CxAD in the older group. We made pilot experiments of the action of full DMSO in the CxAD model and demonstrated its proconvulsant action (data on file). In addition, the proconvulsant action of DMSO was demonstrated in rats exposed to pentylenetetrazol [22]. Saline injection does not modify postictal phenomena.

An agonist of the A1 receptor CCPA in the lower dose partly suppressed the increase in duration of the conditioning AD, whereas the higher dose completely blocked its prolongation in 12-day-old rats, i.e., it suppressed the delayed potentiation; duration of the testing response did not significantly differ from that of the conditioning one after either dose of CCPA, i.e., potentiation is blocked. This might be due to the anticonvulsant effect of CCPA in immature rats [10,12] but also as a tendency to establish postictal refractoriness with a higher dose of CCPA. The postictal refractoriness in 25-day-old animals was not changed by CCPA. In addition to the changes in AD duration, CCPA was able to affect clonic seizures accompanying the second, testing AD—their intensity expressed by Racine’s scale was significantly decreased after the higher dose of CCPA. 

Antagonism of A1 receptors with DPCPX led to extremely long conditioning ADs—delayed potentiation was augmented. It might be explained by the role of adenosine in arrest of seizures [9,10,11]. A detailed analysis of adenosine mechanisms of seizure arrest is presented in a review published by Boison [9]. In addition, DPCPX administration resulted in an increased incidence of mixed ADs, i.e., in the transition of epileptic activity into the limbic system. This effect is probably due to the abolition of adenosine general inhibitory modulation of brain activity mediated by A1 receptors, because under control conditions, this transition was registered only after substantially higher stimulation intensities. Some rats (especially 12-day-old ones) in our older study did not exhibit this transition even after the 15-mA intensity of the stimulation current [23]. The spread of epileptic activity into the limbic system is with high probability realized by thalamic nuclei. Not only limbic (i.e., anterior) nuclei but also mediodorsal nucleus and posterior nucleus might represent the way of spread into limbic structures [24]. The appearance of mixed ADs after the administration of the A1 receptor antagonist suggested the presence of adenosinergic inhibitory modulation in 12-day-old rat pups, i.e., at the early stage of brain maturation.

There was no simple dose–response relationship in groups with DPCPX dissolved in DMSO; the strongest effect was registered after the 0.5-mg/kg dose of DPCPX. The lower efficacy of the 1-mg/kg dose might be explained by an interaction between the drug and solvent (it is supported by the result with DPCPX in suspension) or less probably by the possible presynaptic effect of the high dose partly counteracting the block of postsynaptic A1 receptors, but further analysis has to be carried out. 

The effect of DPCPX on postictal refractoriness is not as clear as that of caffeine (data on file); the reason for this difference may be in the doses used and in combination with the possible action of DMSO. Even a nonsignificant tendency in the experiments with a 50% solution of DMSO complicated the interpretation of effects of DPCPX. The possible synergic action of DMSO and A1 receptor antagonist was demonstrated by quantitatively different results with DPCPX dissolved in DMSO and DPCPX in suspension. The clearly expressed efficacy of caffeine might be also due to a nonspecific action of this antagonist—a simultaneous effect on both types of adenosine receptors in the brain [5,6]. In spite of all these complications, the highest dose of DPCPX was able to partly suppress the postictal refractoriness in 25-day-old rats. A mirror effect of an agonist of A1 receptors CCPA—partial establishment of postictal refractoriness in 12-day-old rats—speaks again for the importance of adenosinergic inhibitory modulation even at this developmental stage. It is in agreement with data on ontogeny of adenosine receptors; A1 receptors are present at birth and their B_max_ in the cortex increases up to postnatal day 21 [25] or 24 [26]. Adenosine uptake [26] demonstrated also an early development of the adenosinergic system. Our data on adenosine A1 receptors in 12- and 25-day-old rats did not fully agree with the results of Geiger et al. [25] and especially with those of Marangos et al. [26]. The difference might be due to methodical reasons—we measured protein, and the older studies were focused on the binding capacity of receptors. It should be necessary to have data on developmental changes of affinity of A1 receptors in the two ages studied in our experiments. The reason for failure of postictal refractoriness in the immature brain might be not only in the overexpression of ionotropic glutamate receptors [27] but also in the immature inhibition of corticothalamocortical circuits generating cortical epileptic afterdischarges. In contrast, the marked effect of the A1 receptor agonist CCPA on pentetrazol-induced generalized tonic–clonic seizures (generated in the brainstem) in 12-day-old rats [12] is in agreement with the caudo-rostral progress of brain maturation. Marked effects of adenosinergic drugs in the younger group are in agreement with our previous studies [10,12] and are in accordance with the higher incidence of A1 receptors in the cortex shown in the present study and in the hippocampus (data on file). 

The possible clinical use of adenosine A1 agonists is limited by the action on adenosine on receptors in other systems (e.g., cardiovascular) and by the fact that adenosine interacts with other neurotransmitter systems as demonstrated for GABA in immature hippocampal neurons [28]. Clinical application might be possible by inhibition of adenosine kinase, the main enzyme catabolizing adenosine [29], or if brain selective ligands for A1 receptors will be accessible (maybe WAG993 or its congeners) [30,31]. 

## 4. Materials and Methods

### 4.1. Animals

Male rats of the Wistar strain were used. All procedures involving animals and their care were conducted according to the ARRIVE guidelines in compliance with national (Act No 246/1992 Coll.) and international laws and policies (EEC Council Directive 86/609, OJ L 358, 1, 12 December 1987; Guide for the Care and Use of Laboratory Animals, U.S. National Research Council, 1996). Adenosine A1 receptors were studied by Western blot analysis during postnatal development (a total of 160 animals) with the focus on two age groups—12- and 25-day-old animals. These two age groups were used for electrophysiological experiments performed in the Institute of Physiology. Sample size was determined in advance according to previous experience with used model and followed the principles of the three Rs (Replacement, Reduction, and Refinement; https://www.nc3rs.org.uk/the-3rs last version with explanation July 2020). Outcome measures and statistical tests were prospectively selected. At beginning of each experimental session, individual animals (four or five rats in one day) were tested (righting, placing, and suckling reflexes) and the animals succeeding in these tests were randomly allocated to two dose groups. All efforts were made to minimize the suffering of baby rats (mainly maintaining their body temperature). Outcome measures were selected prospectively. Data acquisition and analysis were carried out blindly to the treatment. Average body weight (+SD) for P12 animals was 31.19 ± 1.66 g, and for P25 rats, it was 69.80 ± 2.5 g. 

### 4.2. Western Blot Analyses

Western blot analysis was performed to detect changes in adenosine A1 receptor in nine naive age groups (7-, 10-, 12-, 15-, 18-, 21-, 25-, 32-, and 52-day-old rats). Cortical tissue from 54 rats (6 animals/group) was collected for three weeks. The tissue was frozen and stored at −80 °C for 4–5 months before analyses. Mixed samples were prepared from all 6 animals per group using a glass homogenizer with a power-driven Teflon pestle (Helidolph–RZR2021) with 10 mM PBS (pH 7.4) at a 1:4 ratio and protease inhibitor cocktail (# P8340, Sigma-Aldrich, St. Louis, MO, USA). The homogenates were centrifuged (#120951, Sigma-Aldrich, St. Louis, MO, USA, 2–16 PK) at 1000× *g* for 10 min at 4 °C, and approximately 4 mL of the supernatant was collected for analyses. Small volume of the cortical lysate (100 uL) was used for quantification of protein concentration by Lowry’s method [32] with Peterson’s modification [33]. Before electrophoresis, the samples were mixed at a 1:2 ratio with Laemmli loading buffer (#161-0737, Bio-Rad, Hercules, CA, USA) and heated for 20 min at 70 °C for protein reduction. 

Critetion TGX Stain-free gradient gels (8–16%, 18 well, # 567-8104, Bio-Rad) were used for protein separation and protein labeling by binding of trihalo compound to tryptophan residues. After electrophoresis (300 V, 270 mA, 23 min), all gels were activated and visualized by UV light for 5 min by a ChemiDoc™ Touch Imaging System (Bio-Rad, Hercules, CA, USA). The samples were subsequently transferred to nitrocellulose membranes (#170-4271, Bio-Rad, Hercules, CA, USA) using a Trans-blot Turbo apparatus (Bio-Rad). The quality of transfer and volume of protein on the membrane were determined by a ChemiDoc™ Touch Imaging System (Bio-Rad, Hercules, CA, USA). 

Membranes were blocked in 5% nonfat milk in Tris-buffered saline (TBS) for one hour at room temperature and were then incubated overnight at 6 °C with primary antibodies against A1 receptor (1:3000; PA1-041A, Thermo Fisher Scientific, Waltham, MA, USA). The following day, membranes were washed 3 × 10 min in TBS and then incubated for one hour at room temperature with secondary antibody (1:30,000; #211-032-171, Jackson ImmunoResearch Laboratories, West Grove, PA, USA) and washed in TBS, as described above. The chemiluminescent substrate Supersignal WestFemto (#34096, Thermo Scientific) was used for visualization of the protein with the ChemiDoc™. The bands were detected and analyzed with ImageLab software (version 6.1) (pages of the company Bio-Rad, Hercules, CA, USA). After chemiluminescence detection of the target A1 receptor, the membranes were washed for 15 min to remove bound primary and secondary antibodies from the membranes using Restore PLUS Western Blot Stripping Buffer (#46430; Thermo Fisher Scientific, Waltham, MA, USA). These membranes were again washed in TBS buffer and for one hour blocked in 5% nonfat milk in TBS (again at room temperature). Stain-free images of total protein were used to normalize the target protein as a loading control. Western blotting normalization with stain-free technology is comparable to other total protein staining methods (PonceauS, Coomassie Blue, etc.) [34,35]. 

The analysis of the developmental profile of the A1 receptors was focused on the two age groups examined electrophysiologically, i.e., 12- and 25-day-old rats. 

### 4.3. Preparation for Electrophysiology

**Figure 10:**
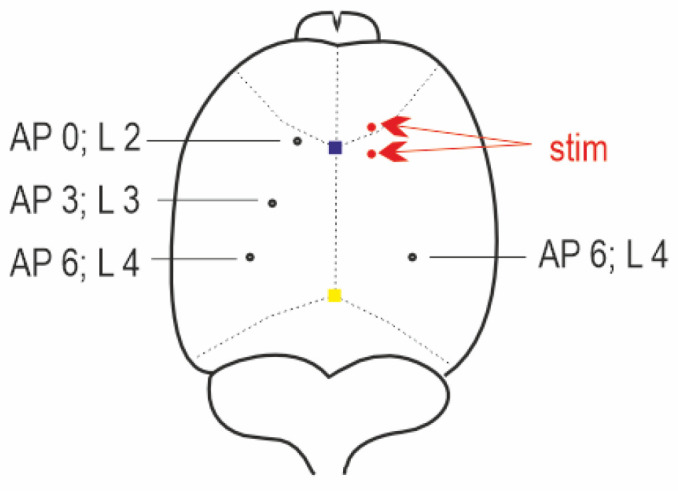
Schematic drawing of placement of electrodes. Blue square = bregma, yellow square = bregma. Stimulation electrodes in red, registration electrodes in black.

Cortical stimulation and registration flat silver electrodes were implanted epidurally under ether anesthesia. This anesthesia was used because of its short duration and safety concerning the survival of immature rats. Stimulation electrodes were placed over sensorimotor area of the right hemisphere (AP −1 and +1, L 2.5 mm), and registration electrodes were placed over left sensorimotor (AP 0, L 2.5 mm), parietal and occipital areas, and over right occipital area. Coordinates for parietal and occipital electrodes were calculated from adult values (AP 3, L 3, and AP 6, L 4 mm, respectively, Figure 10) on the basis of a ratio of bregma–lambda distance (on the average 8 mm in adult rats, 4 mm in 12-day-old animals), therefore coordinates in our group of the younger rats represented half of adult data. Recording from the left frontal region was used for measurement of AD duration, and the other three electrodes served for checking of the spread of epileptic activity. The electrode assembly was fixed to the skull with fast curing dental acrylic. The surgery lasted 10–12 min, and then, the animals were allowed to recover for at least one hour, righting, placing, and suckling reflexes were checked, and only then, the experiments started. Only animals demonstrating these three reflexes formed the presented groups; those not performing these reflexes were eliminated. The animals with implanted electrodes could be used for one experiment only because of a rapid growth of the brain and skull. 

### 4.4. Stimulation and Recording

Stimulation paradigm was the same as in previous publication [21]. Stimulation series lasting 15 s formed by 1-ms biphasic rectangular pulses were generated by a stimulator with a constant current output. To be sure that the first conditioning stimulation elicits an AD, suprathreshold intensity of stimulation current was used—it was 3 mA in the older group; 12-day-old rats needed higher intensity—usually 6 mA. Stimulation was repeated four times, two pairs were applied. An interval between the end of the first, conditioning afterdischarge (AD) and beginning of the second, testing stimulation was 1 min. Immediately after the end of the testing AD (or after the end of stimulation if AD failed to appear), drugs were injected, and 10 min later, the paired stimulation was repeated, i.e., each animal served as its own control comparing pre- and post-drug pairs. Duration of ADs was measured, and ADs pattern and behavior during ADs were evaluated. 

### 4.5. Drugs

One mg of CCPA (2-chloro-*N*^6^-cyclopentyladenosine, Abcam, Cambridge, UK), a selective A1 receptor agonist, was put into suspension with a drop of Tween 80 (approximately 0.02 mL) and was put into 1 mL of water. The doses of 0.5 and 1 mg/kg were administered intraperitoneally. DPCPX (8-cyclopentyl-1,3-dipropylxantine, Abcam, Cambridge, UK) a selective A1 receptor antagonist was dissolved in 50% dimethylsulfoxide in concentration of 1 mg/mL and administered in doses of 0.1, 0.5, and 1 mg/kg i.p. In addition, a suspension of DPCPX (again 1 mg of DPCPX with a drop of Tween 80 was dissolved in 1 mL of water) in doses of 0.5 and 1 mg/kg was used to avoid the effect of the organic solvent dimethylsulfoxide. The doses were chosen on the basis of our previous study of action of these two drugs in a model of pentylenetetrazol-elicited seizures [12]. 

Seven to thirteen animals successfully passing neurological tests formed age, drug, and dose groups. The total number of rats in electrophysiological experiments was 160. 

### 4.6. Statistics

Statistical analyses of adenosine A1 receptor density were performed by unpaired Student’s *t* test using GraphPad Prism 5 software (GraphPAD software). Data are presented as normalized intensity units. 

Western blot analysis was evaluated in National Institute of Mental Health, whereas electrophysiological experiments were performed and evaluated in Institute of Physiology. These institutions possess different versions of GraphPad Prism software. 

Sample size was determined in advance according to previous experience with the given models and followed the principles of the three R’s (Replacement, Reduction, and Refinement; https://www.nc3rs.org.uk/the-3rs last version with explanation in July 2020). Outcome measures and statistical tests were prospectively selected. At the beginning of the study, a simple randomization was used to assign each animal to a particular treatment group. Data acquisition and analysis were carried out blinded to the treatment. Data were analyzed using GraphPad Prism 8 (GraphPad Software, United States) software. Outliners were identified with ROUT test (Q = 1%). Using the D’Agostino–Pearson normality test, all data sets were first analyzed to determine whether the values were derived from a Gaussian distribution. Statistical analyses of adenosine A1 receptor density were performed by unpaired Student´s t test. Data are presented as relative ratio between PD 12 and PD 25 animals. Results of PD12 group were taken as 100%.

Differences in duration of epileptic afterdischarges (C1 vs. T1, T1 vs. C2, C1 vs. C2, and C2 vs. T2) were analyzed using ordinary RM one-way ANOVA followed by Sidak post hoc test. The level of significance was set at 5%. 

In the figures, the data are presented as box plots (from minimum to maximum) and for individual animals are shown as circles.

## 5. Conclusions

CCPA, an agonist of adenosine A1 receptors, shortened cortical epileptic afterdischarges (suppressed delayed potentiation) in both age groups. The immediate postictal potentiation in 12-day-old rats was not present in the second pair of controls, as well as CCPA stimulations. An antagonist (DPCPX) of adenosine A1 receptors augmented the delayed postictal potentiation, but the immediate postictal potentiation was suppressed in 12-day-old animals. If applied in suspension, a tendency to postictal depression was seen.

The possible clinical potential of drugs affecting the adenosinergic system is limited by many peripheral effects (e.g., cardiovascular ones). The way of solving this problem might be the inhibition of adenosine kinase, i.e., an enzyme which catabolizes adenosine and thus increases the adenosine level at sites where it was released [29,30,35,36,37,38], as a promising method for clinical use, or partial agonists of A1 adenosine receptors devoid of effects on other systems like WAG 994 (for review [30,39]). 

## Data Availability

Data is contained within the article, original data and statistics are in the Institute of Physiology CSAS.
